# Talker variability in audio-visual speech perception

**DOI:** 10.3389/fpsyg.2014.00698

**Published:** 2014-07-16

**Authors:** Shannon L. M. Heald, Howard C. Nusbaum

**Affiliations:** Department of Psychology, The University of ChicagoChicago, IL, USA

**Keywords:** talker normalization, talker variability, audio-visual speech perception, multisensory integration, speech perception

## Abstract

A change in talker is a change in the context for the phonetic interpretation of acoustic patterns of speech. Different talkers have different mappings between acoustic patterns and phonetic categories and listeners need to adapt to these differences. Despite this complexity, listeners are adept at comprehending speech in multiple-talker contexts, albeit at a slight but measurable performance cost (e.g., slower recognition). So far, this talker variability cost has been demonstrated only in audio-only speech. Other research in single-talker contexts have shown, however, that when listeners are able to see a talker’s face, speech recognition is improved under adverse listening (e.g., noise or distortion) conditions that can increase uncertainty in the mapping between acoustic patterns and phonetic categories. Does seeing a talker’s face reduce the cost of word recognition in multiple-talker contexts? We used a speeded word-monitoring task in which listeners make quick judgments about target word recognition in single- and multiple-talker contexts. Results show faster recognition performance in single-talker conditions compared to multiple-talker conditions for both audio-only and audio-visual speech. However, recognition time in a multiple-talker context was slower in the audio-visual condition compared to audio-only condition. These results suggest that seeing a talker’s face during speech perception may slow recognition by increasing the importance of talker identification, signaling to the listener a change in talker has occurred.

## INTRODUCTION

In perceiving speech, we listen in order to understand what someone is saying as well as to understand who is saying it. Although the message changes more often in a conversation, there can also be changes between speakers that are important for the listener to recognize. A change in talker can pose a perceptual challenge to a listener due to an increase in the variability of the way acoustic patterns map on to phonetic categories – a problem of talker variability. For different talkers, a given acoustic pattern may correspond to different phonemes, while conversely, a given phoneme may be represented by different acoustic patterns across different talkers (Peterson and Barney, 1952; Liberman et al., 1967; Dorman et al., 1977). For this reason, the speaker provides an important context to determine how acoustic patterns map on to phonetic categories (cf. Nusbaum and Magnuson, 1997). Additionally, a change in talker may be important to recognize given that a listener’s interpretation of a message may depend not just on the speech style of a speaker, but on the attributions about who the speaker is as well (Thakerar and Giles, 1981). For example, indirect requests are understood in the context of a speaker’s status (Holtgraves, 1994). More directly relevant to speech perception however, a listener’s belief about the social group to which a speaker belongs can significantly alter the perceived intelligibility of a speaker’s speech (Rubin, 1992). Additionally, dialect (Niedzielski, 1999) and gender (Johnson et al., 1999) expectations can meaningfully alter vowel perception, highlighting that social knowledge about a speaker can affect the relatively low-level perceptual processing of a speaker’s message, much in the same way that knowledge of vocal tract information can (Ladefoged and Broadbent, 1957; although see Huang and Holt, 2012 for an auditory explanation of the mechanism that could underlie this).

In general there have been two broad views regarding how talker information is recognized. One account, called “talker normalization” (Nearey, 1989; Nusbaum and Magnuson, 1997), suggests that listeners use talker information to calibrate or frame the interpretation of a given message in order to overcome the considerable amount of uncertainty (e.g., acoustic variability, reference resolution, etc.) that arises from talker differences. This view has emerged from an attempt to address the lack of invariance problem through the use of talker-specific information either derived from the context of prior speech (Joos, 1948; Ladefoged and Broadbent, 1957; Gerstman, 1968) or cues within the utterance (e.g., Syrdal and Gopal, 1986). The sufficiency of such models has been demonstrated for vowel perception (e.g., Gerstman, 1968; Syrdal and Gopal, 1986) for both types of approaches. Further, perceptual evidence has come from demonstrations of better recognition for speech from a single-talker compared to speech from different talkers (e.g., Creelman, 1957; Nearey, 1989) and that specific acoustic information can aid in normalizing talker differences (e.g., Nusbaum and Morin, 1992; Barreda and Nearey, 2012).

An alternative view regarding how talker information is recognized suggests that talker information is not used in direct service of message understanding but for source understanding. This view treats the identification of the talker as separate from the process of message comprehension (Pisoni, 1997; Goldinger, 1998). Traditionally, speech perception has been described as a process whereby linguistic units (e.g., phonemes, words) are abstracted away from the detailed acoustic information that is putatively not phonetically relevant. The idea that acoustic information about a talker might be viewed as noise in relation to the canonical linguistic units upon which speech perception relies, has led to the assumption that talker information is lost during this process (e.g., Joos, 1948; Summerfield and Haggard, 1973; Halle, 1985; McLennan and Luce, 2005)^1^. However, the need for preserving talker-specific information for other perceptual goals (Thakerar and Giles, 1981; Holtgraves, 1994), along with evidence suggesting that the perceptual learning of speech is talker-specific (Goldinger et al., 1991; Schacter, 1992; Pisoni, 1993; Nygaard et al., 1994) prompted researchers to adopt a talker-specific view of speech perception.

In the talker-specific view, auditory representations of utterances are putatively represented in a more veridical fashion. As such, both the indexical source auditory information is maintained along with any phonetically relevant auditory information (e.g., Goldinger, 1998). While this view does separately preserve talker-specific auditory information such as fundamental frequency within the auditory-trace, the model has no implications for the representation or processing of other aspects of talker information such as knowledge about the social group of the talker, the dialect of the talker, or the gender of the talker. Further, the echoic encoding account does not explain how talker-specific information that is not in the acoustic channel affects speech processing, as it focuses on the memory representation of auditory patterns.

A number of studies have demonstrated that in a variety of learning situations, variability is important in developing robust perceptual categories that can benefit recognition in diverse listening conditions. In particular, variability in talker has been shown to benefit the long-term memory representations of speech that can facilitate recognition when there is noise or degraded signal or in learning a foreign contrast (Logan et al., 1991; Nygaard et al., 1994; Zhang et al., 2009). However, these studies tend to focus on the benefits of variability in the learning process during which phonetic representations or lexical representations are formed for use in recognition. But beyond this variability in the process of learning speech representations, there is also variability in the moment when one talker stops speaking and another starts. This kind of variability has a short-term effect of slowing recognition, shifting attention to different acoustic properties and increasing activity consistent with an attentionally demanding process (Mullennix and Pisoni, 1990; Nusbaum and Morin, 1992; Wong et al., 2004; Magnuson and Nusbaum, 2007). The difference in these two kinds of situations is not simply that the goal of one set of studies is learning (learning a talker or phonological or lexical forms) vs. speeded recognition, but also that the studies of learning are not designed to evaluate the nature of processing that occurs in the first 10 ms of encountering a new talker but instead focus on the nature of the representations ultimately developed. However, as has been discussed for many decades from Ladefoged and Broadbent (1957) to Barreda and Nearey (2012), variability in the mapping between acoustic patterns and linguistic categories differs across talkers and this variability has been shown to elicit worse performance across a number of measures [slower response times (RTs), lower hit rate, or higher false alarm rate; Wong et al., 2004; Magnuson and Nusbaum, 2007]. Further, the evidence that these performance costs are not mitigated by familiarizing listeners with the talkers (Magnuson et al., 1994) suggests that there is a clear separation between talker variability effects on the short-term accommodation to speech and learning effects in a multi-talker context.

While familiarity with a talker does not appear to influence the talker variability effect found in the short-term accommodation to speech, it remains unclear whether non-acoustic information about a talker can moderate the effect of talker variability. Much of the research regarding talker variability effects has examined the notable acoustic variability found in a multiple-talker context. However, a multiple-talker context can produce variability in other sensory channels (beyond the acoustic), which could impair talker identification and message comprehension. Given that conversations can take place among several interlocutors in a face-to-face context, it is reasonable to ask how the presence of face information affects speech perception when the talker changes. If watching a talking face provides cues for both talker identification and message comprehension there are two potential effects. One possibility is that seeing a new talker will slow recognition, as it will prompt the listener to enter into an attention-demanding (Nusbaum and Morin, 1992; Wong et al., 2004) process by which the speech of the new talker is perceptually normalized (Nearey, 1989; Nusbaum and Magnuson, 1997). Conversely, the presence of face information may speed up recognition by providing a converging source of phonetic information through visemes that allows the listener to achieve faster and/or more accurate word recognition (Sumby and Pollack, 1954; Summerfield, 1987; Massaro and Cohen, 1995; Rosenblum et al., 1996; Lachs et al., 2001).

Previous research has demonstrated that a person’s face is an important source of information about social category membership, which can also influence speech perception. As noted already, the subjectively rated intelligibility of the same speech signal is different depending on whether the speech is accompanied by pictures of putative speakers from different racial groups (Rubin, 1992). Similarly, the classification of vowels can be changed by seeing a different gendered face presented falsely as the speaker (Johnson et al., 1999). In both cases, participants simply viewed static photographs that identified the speaker. Given human face expertise (e.g., Diamond and Carey, 1986; Gauthier and Nelson, 2001), observers are very accurate in recognizing faces (Bahrick et al., 1975), even more so than in recognizing voices (Read and Craik, 1995; Olsson et al., 1998; Wilding and Cook, 2000). Thus, the presence of visual face information provides an ecologically reliable cue about speaker identity. Work by Magnuson and Nusbaum (2007) has demonstrated that the effect of talker variability can be mediated entirely by expectations the listener holds regarding talker differences. This study showed that when an acoustic difference (a small F0 difference) was attributed to normal production variability of a single-talker, variation in F0 did not slow recognition down any more than a constant F0. However, when the identical acoustic difference was interpreted (based on prior expectation) as a talker difference, the same F0 variability led to slower recognition compared to a condition with a constant F0. This demonstrates that it is not the acoustic variability that slows recognition but the knowledge of what that variability means. Seeing a face change provides similar knowledge to listeners, as it signals to the listeners that a change in talker has indeed occurred. Therefore, it is reasonable that visual face information may act to signal a change in talker and therefore the need to calibrate perception through normalization.

While there is evidence that a still photograph can give clear information about the identity of a speaker, a video of the speaker’s face provides additional information, as a talking face can additionally show visible articulatory gestures. For example, the intelligibility of speech in noise (Sumby and Pollack, 1954) as well as speech heard through cochlear implants (Goh et al., 2001; Lachs et al., 2001) is significantly improved by additionally seeing a speaker talk. However, there is clear evidence that the visual information of mouth movements is not simply redundant with the speech signal. The McGurk and MacDonald (1976) effect clearly demonstrates that independent articulatory information can be visually gleaned and integrated with speech signals during perception. To engender the McGurk and MacDonald (1976) effect, a participant is shown a video of a mouth producing one place of articulation (e.g., /ka/) while hearing acoustic information corresponding to a different place of articulation at the same time (e.g., /pa/). This presentation combination results in the perception of a third illusory place of articulation (e.g., /ta/). Indeed, using neuroimaging during the presentation of McGurk stimuli, Skipper et al. (2007) demonstrated that the pattern of brain activity in the supramarginal gyrus starts out consistent with the acoustic information (e.g., /pa/) but changes over time to be consistent with the final percept (i.e., /ta/), whereas brain activity in the middle occipital gyrus starts out consistent with the visual mouth movements (e.g., /ka/) but ends up responding with a pattern consistent with the final percept. However, the ventral premotor region starts out coding the perceptual category and maintains that activity pattern. The illusion along with the neuroimaging data suggests that different sensory systems initially code different sources of perceptual information about speech in interaction with divergent information represented in the motor system. If seeing mouth movements improves recognition performance as shown behaviorally by recruiting premotor cortex and increasing superior temporal activity (Skipper et al., 2005, 2007), it is possible that slower recognition and/or worse accuracy associated with a change in talker might be ameliorated if not eliminated, given that seeing mouth movements may provide additional information such as visemes that could be used to limit or constrain phonetic interpretation from the acoustic channel.

Thus seeing a talker can visually provide both message-relevant and source-relevant information, just as the acoustic pattern of an utterance does. On the one hand, a face can convey clear talker identity information to an observer, which can be important when listening to speech because it may signal a change in talker and the need to calibrate perception through normalization. On the other hand, mouth movements can additionally convey articulatory information that may help constrain acoustic variability. Although Olsson et al. (1998) have shown that speech is a much more effective cue to message content than mouth movements, Rosenblum et al. (1996) have demonstrated that even with the low accuracy of lip reading, this information significantly boosts the recognition of spoken words in noise. Given these two different possibilities for the way that visual information is used by listeners, it is unclear how seeing talkers would affect speech recognition when there is talker variability. Visual talker information could act as a strong signal of talker change (thereby requiring more perceptual analysis of the face and speech) ultimately slowing speech recognition. Conversely, the presence of a face could speed up recognition through the provision of concurrent viseme information.

The present study was carried out to address how seeing a talker would influence speech recognition in a multiple-talker context. Listeners performed a speeded word recognition task, listening for spoken words that were designated as a target. Targets differed in several phonemes from other targets and distracters to ensure that recognition did not depend on a single phonetic contrast. Listeners were required to respond every time they recognized a target. On each trial, four occurrences of a target word were presented randomly in a sequence along with 12 randomly selected distracters. On single-talker trials, one talker produced all the target and distracter speech, while in multiple-talker trials, multiple-talkers produced both targets and distracters. In the present study, one group (half of the participants) was presented with only the acoustic speech signal. This portion of the study replicates the design of previous, audio-only talker variability studies using speeded target detection (e.g., Nusbaum and Morin, 1992; Wong et al., 2004; Magnuson and Nusbaum, 2007). A second group (half of the participants) was presented with audio-visual speech in which the listener could see and hear the talker producing the utterance. Previous, audio-only, talker variability studies have demonstrated better performance (fast reaction times, higher hit rate, or lower false alarm rate) for single-talker trials compared to multiple-talker trials (Wong et al., 2004; Magnuson and Nusbaum, 2007).

There are two possible predictions regarding the way that seeing a talker will influence speech recognition speed in the present study. If seeing a talker’s mouth movements provides viseme information to reduce acoustic-phonetic uncertainty, then audio-visual speech will have better performance than audio-only speech, independent of how much talker variability is present. Further, viseme information present when seeing a talker could also reduce, if not eliminate the poorer recognition performance associated with talker variability. Performance in the multiple-talker condition could be improved if viseme information constrains the one-to-many mapping of acoustic segments onto phonetic categories. If this is the case then recognition performance for single-talker trials should not significantly differ from recognition performance for multiple-talker trials in the audio-visual condition. Indeed, the poorer performance found in multiple-talker trials in audio-only studies may be an artifact of the “unnatural” (in the context of evolution) situation of hearing speech without seeing the talkers.

Another possible prediction however, is that seeing talkers may be a much more powerful signal of talker identity than simply hearing speech. If so, then seeing talkers might result in even poorer performance than has been found in multiple-talker trials compared to single-talker trials, if the face acts as a cue for listeners to enter into a talker normalization process. If this is the case then both audio-only and audio-visual speech should both show poorer performance in the multiple-talker condition when compared to single-talker condition. Further, if the presence of the face does act as a more effective cue to talker change, then the multiple-talker condition might show even poorer performance in audio-visual condition compared to audio-only condition. This would be the case if audio-only speech is a less effective cue to talker change than audio-visual speech and as such, results in producing more occurrences of talker normalization in the audio-visual condition. As poorer performance could manifest as an increase in reaction time, a decrease in hit rate, an increase in false alarm rate or a drop in d-prime, every participant’s average RT, hit rate, false alarm rate, and d-prime were measured for each condition.

## MATERIALS AND METHODS

### PARTICIPANTS

Forty-six participants (31 female) were recruited from the University of Chicago undergraduate community and were between 18 and 26 years of age. One participant was dropped from analysis due to a technical problem in collecting data, and a further participant was excluded from analysis due to reported excessive fatigue (her overall accuracy was 79%). Both of the excluded participants were female. All of the participants were native speakers of American English, with no history of hearing, speech, or vision disorders reported. Participants were compensated with course credit and were debriefed upon the conclusion of the experimental session. Additionally, informed consent, using a form approved by the University of Chicago Institutional Review Board, was obtained from all subjects.

### STIMULI

The stimuli consisted of audio-visual and audio-only versions of the same recordings of words, produced by three talkers, as different groups of listeners performed speeded word recognition for different pairs of speakers. Specifically, half of the participants performed the speeded word recognition with speech from two male talkers (Talker CL and Talker SH), while the other half of participants performed the speeded word recognition with speech from a male and a female talker (the same stimuli by Talker SH were used again, and Talker CL was replaced by Talker SK, a female talker). This was done so as to ensure that any differences we found were not due to a particular pair of speakers. The words used as stimuli were selected from the Harvard phonetic-balanced word list (IEEE Subcommittee on Subjective Measurements, 1969). We selected the words used by Magnuson and Nusbaum (2007), namely: “ball,” “bluff,” “cad,” “cave,” “cling,” “depth,” “dime,” “done,” “gnash,” “greet,” “jaw,” “jolt,” “lash,” “knife,” “park,” “priest,” “reek,” “romp,” and “tile.” Of these 19 words, “ball,” “cave,” “done,” and “tile” were used as target words. The stimuli were produced by all three speakers in front of a neutral green screen. The video recording was made with a Canon GL-1 digital camcorder. The visual portion of the stimuli consisted of the speaker’s face directly facing the camera. The size of each talker’s face was equalized across all of that talker’s stimuli. Additionally, the relative differences in face size were maintained between the two speakers.

High-quality sound recordings (32 kHz, 16 bit) were simultaneously recorded along with the video using an Alesis ML-9600 sound recorder. The high-quality sound recordings were then used to replace the original soundtrack from the audio-visual recording using Finalcut Pro. The audio component of all the stimuli were RMS normalized to an average of 57.2 dB SPL. The duration of each word (from sound onset to sound offset) was measured, and the durations of words (both in terms of video and sound) produced by Talker CL and Talker SK were shortened to match the duration of each corresponding word produced by Talker SH as Talker SH had the shortest durations. Duration changes for the sound portion were accomplished by applying the PSOLA algorithm in Praat (Boersma, 2001). PSOLA was also applied to the stimuli produced by Talker SH with the speed factor of 1, as a control. Duration changes for the video portion were accomplished by altering the speed of the video in Finalcut Pro. Given that duration changes were identical for both audio and visual aspects of the recording, the final audio-visual presentation sounded natural and was free from any asynchrony. In order for the stimuli to be short enough for use in a speeded target-monitoring task, the stimuli were edited down to a length of 666 ms. In order to keep the audio portion of the audio-visual and audio-only stimuli comparable and to match stimulus durations (AV and A) across conditions, all the stimuli were edited to begin at the start of sound onset. While previous research on the time course of audio-visual speech perception has indicated that some visual cues can precede the acoustic onset by 80–100 ms (Smeele, 1994, Unpublished Doctoral dissertation; Munhall and Vatikiotis-Bateson, 1998), a gating study by Munhall and Tohkura (1998) suggests that the visual information that precedes the acoustic onset is not necessary to see a significant contributions of visual information in speech perception. Further, pretesting indicated that the stimuli were perceived as natural productions with no unnatural changes, asynchronies, or jump-cuts perceived. As such, the audio-only stimuli were equivalent to the audio-visual stimuli, except that the video channel was stripped from the audio-visual stimuli.

### PROCEDURE

**FIGURE 1:**
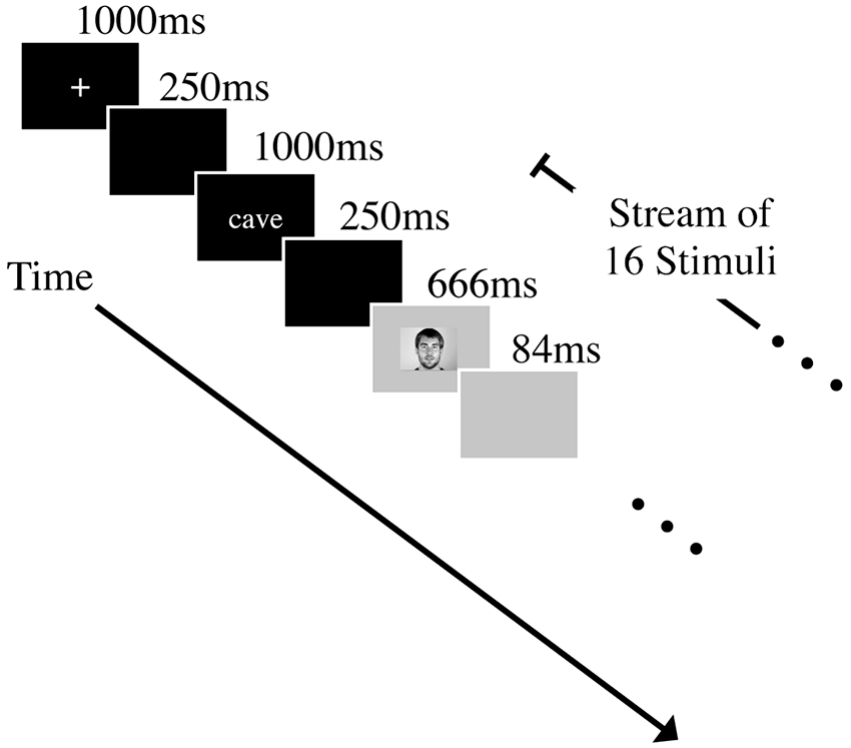
**Experimental format of an audio-visual trial.** Each trial started with a fixation cross that was presented at the center of a black screen for 1000 ms. This was followed by a blank, black screen for 250 ms. Participants were then shown a printed target word (ball, done, cave, or tile) for 1000 ms. Another 250 ms pause preceded the presentation of the spoken stimuli. A stream of 16 spoken words was shown on each trial. Each stimulus was 666 ms, followed by a silent blank screen for 84 ms before the next stimulus was presented. Four word targets were pseudo-randomly placed at ordinal positions between the 1st and 16th stimuli (i.e., positions 2 to 15) such that the targets were separated by at least one distracter. Participants were instructed to press the space bar as quickly and as accurately as possible whenever they recognized the target word. Stimuli either came from only one speaker (the single-talker condition) or from two speakers (the multiple-talker condition) depending on the condition.

The experiments consisted of a speeded target-monitoring task. Before beginning the monitoring task, participants were informed that an orthographic form of a target word would be presented before every trial and that, depending on the modality condition, a sequence of audio, or audio–video recordings of spoken words would follow. Participants were instructed to press the space bar as quickly and as accurately as possible whenever they recognized the target word. At the beginning of each trial, a fixation cross was presented at the center of a black screen for 1 s. A blank black screen was then presented for 250 ms before the printed target word (for 1 s). Another 250 ms pause preceded the presentation of the spoken stimuli. A stream of 16 spoken words was presented for each trial; each stimulus was 666 ms, followed by a silent blank screen for 84 ms before the next stimulus was presented (total SOA 750 ms). Four word targets were pseudo-randomly placed at ordinal positions between the 1st and 16th stimuli (i.e., positions 2 to 15) such that the targets were separated by at least one distractor. On each trial, one target was chosen from the set “ball,” “done,” “cave,” and “tile.” Twelve distracter words were randomly selected from the full set of stimuli, excluding the designated target (see **Figure 1**). After one practice trial, a block of 12 test trials followed, all with either stimuli from only one speaker (the single-talker condition) or from two speakers (the multiple-talker condition). In the latter condition, the talker for each of the 16 words in a trial was randomly determined. Each possible target word appeared as the target for three trials within each of four different conditions, and the order of which target was selected for a particular trial was randomized. Each participant received all four of the talker conditions (single-talker 1 condition, single-talker 2 condition, and multiple-talkers conditions combining the two talkers). Participants received either audio-visual or audio-only stimuli depending on what modality condition to which they were assigned. Every participant’s RT, hit rate, false alarm rate, and d-prime were measured. Participants were always explicitly informed (both verbally and by printed instructions) of the identity of each talker condition before they began trials in that condition.

## RESULTS

**FIGURE 2:**
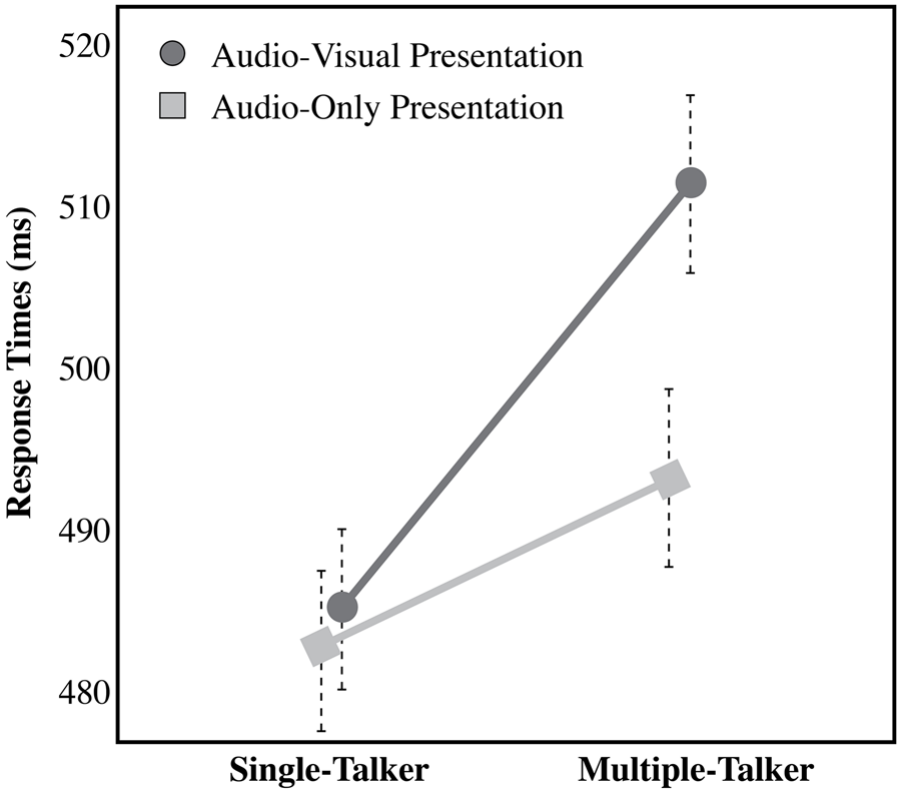
**Response times (RTs) for the single-talker and multiple-talker conditions for both presentation modalities (audio-only and audio-visual).** Error bars represent 1 SE.

In order to examine the effect of audio-visual information on the talker variability cost, a split plot analysis of variance (ANOVA) was carried out [Talker Variability (Single-Talker vs. Multiple-Talker) × Modality of Presentation (Audio-only vs. Audio-visual), with Talker Variability as the within-subject factor and Modality of Presentation as a between-subject factor], for the dependent measures of RT, hit rate, false alarm rate, and d-prime. For the dependent measure of RT, a significant main effect of Talker Variability was found, indicating that listeners are faster to recognize speech from a single-talker (484 ms ± SEM) than from multiple-talkers [502 ms; *F*(1,42) = 27.75, *p* < 0.001]. A planned comparison indicates that the recognition time is significantly slower in the multiple-talkers trials compared to the single-talker trials in the audio-only condition [*t*(21) = 1.637, *p* = 0.05]. This replicates other audio-only talker variability work that has used this task previously (Wong et al., 2004; Magnuson and Nusbaum, 2007). There was no main effect of Modality of Presentation [*F*(1,42) = 0.494, *p* = 0.48]. A significant interaction effect of Modality × Talker Variability however, reveals that the performance cost between multiple-talker trials and single-talker trials was increased by 15 ms in the audio-visual condition (26 ms) compared to the audio-only condition [11 ms; *F*(1,42) = 5.13, *p* = 0.03]. This interaction effect, as seen in **Figure 2** is clearly driven by RT differences across modalities in the multiple-talker trials (i.e., between the audio-only multiple-talker trials and audio-visual multiple-talker trials), as there is little reaction time difference between the audio-only and audio-visual single-talker trials (mean RT in audio-only for single-talker trials was 482 ms. and mean RT in audio-visual for single-talker trials was 485 ms). Thus, it is unlikely that the interaction effect is due solely to the presence of visual information in the task, as we would have seen a similar delay in the single-talker audio-visual trials, but we did not. For this reason, the increase in RT in the audio-visual trials is likely due to extra talker information in the visual display. The same analyses were carried out using hit rate, false alarm rate, and d-prime^2^ but none of these analyses yielded any significant effects or interactions (see **Table 1** for a summary of results for the DV of false alarm rate, **Table 2** for a summary of results for the DV of hit rate, and **Table 3** for a summary of results for the DV of d-prime.).

**Table 1:** Summary of results from the split plot ANOVA [Talker Variability (Single-Talker vs. Multiple-Talkers) × Modality of Presentation (Audio-only vs. Audio-visual), with Talker Variability as a within-subject factor and Modality of Presentation as a between-subject factor] for the dependent measure of false alarm rates.

Source	*F* statistic	*p*	Estimated means (standard error)
Talker variability	0.409	0.526	0.010 (0.001) single-talker
			0.009 (0.001) multiple-talkers
Talker Variability × Modality of Presentation	2.670	0.110	0.009 (0.002) audio only single-talker
			0.010 (0.002) audio only multiple-talkers
			0.011 (0.002) audio-visual single-talker
			0.008 (0.002) audio-visual multiple-talkers
Modality of presentation	0.011	0.918	0.010 (0.002) audio-only
			0.010 (0.002) audio-visual

**Table 2:** Summary of results from the split plot ANOVA [Talker Variability (Single-Talker vs. Multiple-Talkers) × Modality of Presentation (Audio-only vs. Audio-visual), with Talker Variability as a within-subject factor and Modality of Presentation as a between-subject factor] for the dependent measure of hit rates.

Source	*F* statistic	*p*	Estimated means (standard error)
Talker variability	0.199	0.658	0.964 (0.006) single-talker
			0.962 (0.005) multiple-talkers
Talker Variability × Modality of Presentation	0.797	0.377	0.955 (0.008) audio only single talker
			0.957 (0.007) audio only multiple-talkers
			0.973 (0.008) audio-visual single-talker
			0.967 (0.007) audio-visual multiple-talkers
Modality of presentation	1.897	0.176	0.956 (0.007) audio-only
			0.970 (0.007) audio-visual

**Table 3:** Summary of results from the split plot ANOVA [Talker Variability (Single-Talker vs. Multiple-Talkers) × Modality of Presentation (Audio-only vs. Audio-visual), with Talker Variability as a within-subject factor and Modality of Presentation as a between-subject factor] for the dependent measure of d-primes.

Source	*F* statistic	*p*	Estimated means (standard error)
Talker variability	0.505	0.481	0 4.351 (0.101) single-talker
			4.289 (0.089) multiple-talker
Talker Variability × Modality of Presentation	0.000	0.988	4.282 (0.143) audio only single-talker
			4.221 (0.125) audio only multiple-talkers
			4.420 (0.143) audio-visual single-talker
			4.357 (0.125) audio-visual multiple-talkers
Modality of presentation	0.653	0.423	4.252 (0.120) audio-only
			4.389 (0.120) audio-visual

## DISCUSSION

Visual information showing a speaker’s mouth movements together with speech production has been shown to improve intelligibility of speech under adverse listening conditions (Sumby and Pollack, 1954; Summerfield, 1987; Massaro and Cohen, 1995; Rosenblum et al., 1996; Lachs et al., 2001). Research shows that talker variability hurts recognition accuracy (e.g., Creelman, 1957) and recognition speed (Mullennix and Pisoni, 1990; Magnuson and Nusbaum, 2007) providing what could be viewed as an adverse listening situation. If this impairment of recognition performance is a result of reduced intelligibility due to phonetic uncertainty (cf. Magnuson and Nusbaum, 2007) then converging information about phonetic identity from a speaker’s visemes (Skipper et al., 2005) could improve performance. However, the results show that visual information that is coincident with the acoustic information does not lead to faster recognition in a multiple-talker context; rather the presence of a speaker’s face appears to increase the talker variability effect. Listeners who additionally saw a talker’s face concurrent with hearing a talker were significantly slower to recognize speech in multiple-talker trials compared to single-talker trials and were slowed in this more than listeners who could only heard the speakers. This effect of slowing word recognition for multiple-talker trials when listeners could see each talker however, is not due to the presence of the face alone as there was little difference between audio-only single-talker trials compared audio-visual single-talker trials. For this reason, the exacerbation of the talker variability effect in the audio-visual condition compared to the audio-only condition is not simply a distraction effect of visual information.

The current work only examines the benefits of visual information that is coincident with acoustic information, as all the stimuli across conditions (A and AV) were edited to begin at the start of sound onset. While work by Munhall and Tohkura (1998) demonstrates that visual information is continuously available and incrementally useful to a listener, it is possible that the visual information that precedes the acoustic onset may be helpful in ameliorating the talker variability effect. Work by Smeele (1994, Unpublished Doctoral dissertation) demonstrates that some visual cues can precede the acoustic onset by 80–100 ms. As such, this window may help to prime listeners that a talker change has indeed occurred even before the acoustic signal begins, assuaging the perceptual cost of talker variability. Still, the current work suggests that while visual information that is coincident with acoustic information can influence speech perception (Munhall and Tohkura, 1998), it does not mitigate the short-term accommodation to variability found in a multiple-talker context.

These results are consistent with the perspective that seeing a person speak provides more information about the speaker and the speech than just listening to the speech alone. First, a face conveys clear identifying information, as well as providing information relevant to the message content. Visemes – visual information from mouth shapes (Fisher, 1968) – provide phonetic information, which affects speech perception, and even possess the ability to change what is heard in the acoustic signal as in the McGurk effect. Why does seeing a talker slow recognition even more when there is talker variability? Clearly seeing a talker increases the perception of variability. Even when listeners do not perceive a talker difference in speech (Fenn et al., 2011) seeing the face of a person change in this situation will act as a robust cue that a change in speaker has occurred. When a listener knows that there is a talker change, even when there has been none, there are slowing effects on speech recognition times. Magnuson and Nusbaum (2007) showed that the effect of talker variability is due to the knowledge of a talker change or difference rather than the specifics of an acoustic difference. In the present study, the change in face makes absolutely clear to listeners that there has been a change in talker. In this respect the present results are entirely consistent with previous research.

What is the mechanism by which talker variability interacts with modality? Wong et al. (2004) argued that changes in the talker increased demands on attention in speech processing, showing increased superior parietal activity and increased superior temporal activity. In addition, there was a trend toward increased activity in the premotor system when there was talker variability. Moreover, audio-visual speech perception increases brain activity in the premotor system as well (Skipper et al., 2005). From these results, one could predict that audio-visual talker variability might produce an interaction in activation within perisylvian areas that are involved in speech perception. Such increases in activity might correspond to slower processing rather than faster processing, in that suppression of neural activity by relevant information is usually associated with priming and faster responses (Grill-Spector et al., 2006).

While talker normalization accounts have suggested that slowing due to talker variability is a consequence of using talker vocal characteristics to calibrate phoneme processing in the context of new talker, it has also been suggested that listeners also need to identify talkers for more than just reducing phonetic uncertainty. Labov (1986) has argued that listeners need to understand the social context of a message in order to understand it. For example, Holtgraves (1994) has shown that speech is understood differently depending on the attributed power of the speaker. Rubin (1992) demonstrated that a picture of a putative speaker displaying racial group membership could change the perceived intelligibility of speech. Johnson et al. (1999) have shown that changing expectations about a speaker’s gender, just from a static picture of the speaker, can change vowel perception. Niedzielski (1999) has shown that changing listeners’ beliefs about a speaker’s dialect can change vowel perception. All of these examples reflect the way that knowledge about a speaker’s social identity can change speech perception. Although a speaker’s social identity can be conveyed through speech by dialect or voice differences, seeing a person’s face conveys a great deal more social information. The present results suggest that listeners will process this identifying information even if there is a slight cost in recognition speed, which may reflect the importance of social information in speech understanding.

## Conflict of Interest Statement

The authors declare that the research was conducted in the absence of any commercial or financial relationships that could be construed as a potential conflict of interest.

## Footnotes

1 Although, it is possible that talker information, even under a talker normalization rubric, is preserved in parallel representational structures for other listening goals (e.g., Hasson et al., 2007).

2 To calculate d-prime, a hit rate or false alarm rate of 1 or 0 could not be used to obtain actual z-scores (as probabilities of 1 and 0 would correspond to z-scores of ∞ and -∞, respectively). For this reason, the formula [(*n* ^∗^ 2) ± 1]/(*t* ^∗^ 2), where *n* equals the total number of hits or false alarms, and *t* equals the total number of trials, was used as an approximation.
